# Veterinary Technician Specialists: Perceptions and Experiences Related to VTS Credentials and Skill Utilization

**DOI:** 10.1111/vec.70039

**Published:** 2025-10-07

**Authors:** Lori R. Kogan, Leslie Carter, Kelly Foltz

**Affiliations:** ^1^ Department of Clinical Sciences, College of Veterinary Medicine and Biomedical Sciences Colorado State University Fort Collins Colorado USA; ^2^ BluePearl Tampa Florida USA

**Keywords:** advanced‐level veterinary practitioner, credentialing, veterinary nurse specialist, veterinary technician specialist utilization

## Abstract

**Objective:**

To examine the motivations of veterinary technician specialists (VTSs) in pursuing advanced credentials, the impact of having their VTS credential(s), and their views surrounding the potential barriers to optimal utilization of VTS skill and knowledge.

**Design:**

Electronic survey distributed via veterinary organizations, associations, and social media from December 2023 through May 2024.

**Participants:**

A total of 577 veterinary technician and nurse specialists.

**Setting and Interventions:**

Online survey.

**Measurement and Main Results:**

A total of 577 participants completed the survey. Factor analysis identified four items associated with reasons to become credentialed as a VTS (in descending order of importance): expanded responsibilities, personal growth, recognition/respect, and external influences. Five elements of potential change after earning the VTS credential were also identified (in descending order of prevalence): personal growth, professional recognition/respect, expanded responsibilities, elevated role, and career growth. A total of 76.4% of participants indicated that they had received a pay increase after obtaining the VTS credential; 77.4% reported the increase was ≤10%. A total of 84.2% of participants stated feeling that VTSs are underutilized in the workplace.

**Conclusions:**

The prime motivator for participants to earn the VTS credential was to expand their role and responsibilities; however, many did not experience significant changes in responsibility or scope of clinical practice after receiving their credentials. Most participants felt that VTSs are underutilized, with a lack of role clarity and differentiation from non‐VTS colleagues identified as the largest barrier. There is a clear need for better role clarification and an opportunity for education of both veterinary professionals and clients regarding the role, education, and training of VTSs. Although the majority of VTSs recommend the credential to others, it is unclear whether completion of the VTS leads to a commensurate expansion of responsibilities and improvement in compensation that increase the engagement, retention, and career satisfaction of these technicians/nurses.

**Abbreviations**
ALVP: advanced‐level veterinary practitioner
AVECCTN: Academy of Veterinary Emergency and Critical Care Technicians and Nurses
CrVT/N: credentialed veterinary technician/nurse
DVM: veterinarian
VNS: veterinary nurse specialist
VTS: veterinary technician specialist

## 1 Introduction

Credentialed veterinary technicians/nurses (CrVT/Ns) are foundational to a hospital's ability to provide high‐quality medicine and optimal patient care. In the United States, CrVT/Ns are most commonly individuals who have graduated from a 2‐ or 4‐year veterinary technology degree program accredited by the American Veterinary Medical Association Committee on Veterinary Technician Education and Activities and have successfully passed the Veterinary Technician National Examination or foreign equivalent. In countries that recognize the veterinary nurse title, CrVT/Ns may choose to pursue advanced credentials as a veterinary technician specialist (VTS) or veterinary nurse specialist (VNS).

The Academy of Veterinary Emergency and Critical Care Technicians and Nurses (AVECCTN) was the first organization to be recognized by the National Association of Veterinary Technicians in America as a veterinary technician specialty academy. Academy requirements differ, but all are demanding and involve substantial investments of time and energy. Most require some variation of the following: documented clinical experience or hours worked in the area of specialty medicine, skills verification, case logs, case reports, and letters of recommendation. Once these items are reviewed, candidates receive permission to sit for the academy's comprehensive examination [1].

Given the rigor of the requirements, it is perhaps unsurprising that while there are an estimated 70,000 CrVT/Ns in the United States [2], there are only approximately 1500 VTSs^1^, with limited research focused on this population. Previous studies have centered around compensation, with the exception of a single study available on career satisfaction [3, 4, 5]. One study conducted in 2013 found that the typical VTS was a White woman between 25 and 45 years of age who had been a veterinary technician for 11–15 years and did not receive a bonus for obtaining VTS certification, although she did receive a pay raise [4]. This is similar to data reported by Norkus in 2007 [3]. In terms of job fulfillment and burnout, a recent study comparing veterinary assistants, CrVT/Ns, and VTSs found that VTSs reported higher levels of job fulfillment than veterinary assistants but not CrVT/Ns, and no differences were found in burnout level [6].

Finally, advanced‐level veterinary practitioner (ALVP) roles have been proposed as potential solutions to veterinarian shortages and care deserts in the United States, although the educational, credential, and supervision requirements for an ALVP have yet to be determined. VTSs, with their advanced skill sets and knowledge, were posited to have strong opinions about, and potentially high interest in, such a role.

Despite the significant time and resources invested in becoming a VTS, little is known about why veterinary technicians choose this path and how their careers change after earning their VTS credential. The current study was designed to provide insight by surveying VTSs about their opinions and experiences related to VTS credentialing in an effort to highlight potential areas of opportunity within the profession.

## 2 Materials and Methods

The study was reviewed and classified as exempt by the Colorado State University Institutional Review Board.

An anonymous online survey was created with commercial software^2^ to assess the experiences and opinions of VTSs and VNSs. In the remainder of this paper, the term VTS will be used to encompass both VTSs and VNSs. The voluntary survey was distributed through veterinary‐related organizations, listservs, and social media sites from December 2023 through May 2024.

The survey primarily consisted of Likert scale [7] questions, with free‐text boxes used to gather more detailed information. Demographic questions included whether participants are currently or were previously credentialed as a VTS (yes/no), specialty academy in which participants were VTS credentialed, structure of the practice's ownership (corporation, privately owned, other, unknown), time spent working in veterinary medicine, length of time the participant has been VTS credentialed, whether they have a veterinary‐related master's degree, age, sex, race, ethnicity, and country in which they practice. Those who indicated they were credentialed as a VTS in the past, but not currently, were asked to indicate why. All participants were asked to rate a series of 18 items in terms of their importance to becoming a VTS. Examples included, “Desire to expand my knowledge base and skills,” “Desire to have more autonomy at work,” and “Desire for greater respect.” The next series of items (*n* = 31) pertained to potential results of obtaining VTS credentials. Participants were asked to indicate how true (not true at all, somewhat true, very true) each of the statements were as a result of their VTS credential. Examples included, “I feel like a more integral member of the veterinary medical team,” “My knowledge, skills, and patient care improved,” and “I perform more advanced procedures than I did prior to obtaining my VTS.” Additional questions centered around pay increase (and by what percentage), monetary bonus (yes/no), and, if not already offered, the opportunity for production‐based income (yes/no) or profit sharing (yes/no).

Participants were next asked if (and how) their VTS credential was acknowledged by their practice (e.g., name badge, uniform marking). They were asked if (and how) their hospital educated clients about the roles and responsibilities of VTSs (e.g., hospital TV/videos, posters). The survey also included a series of questions that asked participants to share their perceptions of potential barriers to optimal skill and knowledge utilization of VTSs (no barrier, minimal barrier, moderate barrier, large barrier). Examples included, “Veterinarians' [DVMs]’ perceived lack of trust in VTS advanced skill set,” “Lack of role clarification/differentiation between VTS and non‐VTS duties/skills within the practice,” and “Lack of self‐promotion by VTS.”

Participants were asked if they would recommend pursuing a VTS credential to others, whether they pictured themselves being a VTS 5 years in the future, and how their VTS credential influenced their level of clinical autonomy when performing several specific tasks. For each of these tasks, participants were asked to indicate whether they did not perform the task before VTS credentialing, and whether they still do not perform the task; performed the task before VTS credentialing under direct supervision; performed the task before VTS credentialing with no/minimal supervision; did not perform the task before VTS credentialing but now perform it under direct supervision; or did not perform the task before VTS credentialing but now perform it with no/minimal supervision. Examples of tasks included, “Recommend an initial diagnostic plan to the DVM,” “Perform thoracocentesis,” and “Design anesthetic plans.”

In the final part of the survey, participants were asked a series of questions pertaining to an ALVP position, including their interest in pursuing an ALVP position and how they envision a working relationship between a veterinarian and an ALVP should function.

### 2.1 Statistical Analyses

Descriptive statistics are stated for most questions. One‐way ANOVA was conducted to assess the different reasons for becoming a VTS and interest in becoming an ALVP based on age, as well as differences in the impact of VTS credential(s) based on work setting (e.g., private practice, corporate owned). The number of responses for each question is noted when not all participants answered the question.

Factor analysis was used to identify primary reasons to become VTS credentialed along with potential benefits of the VTS credential. For both analyses, collinearity between the variables and intercorrelation among items was assessed to ensure all items were contributing to the latent construct. Each item was determined to be conceptually distinct with no multicollinearity or singularity issues because no items correlated at a level >0.8. Eighteen items were examined with the Kaiser–Meyer–Olkin measure of sampling adequacy to assess the reasons to become VTS credentialed, and responses were deemed acceptable at 0.94. Bartlett's test of sphericity was significant (*χ* ^2^(465) = 8606.16, *p* < 0.001), and the communalities were all >0.30. Therefore, the 18 items were found to share some common variance with other items and were deemed suitable for factor analysis. Principal axis factor with principal component analysis as the extraction method and Direct Oblimin with Kaiser normalization as the rotation method was used. Similarly, the potential impacts of VTS credentialing were examined using the Kaiser–Meyer–Olkin measure of sampling adequacy and were deemed acceptable at 0.86. Again, Bartlett's test of sphericity was significant (*χ* ^2^(153) = 3862.67, *p* < 0.001), and the communalities were all >0.30. All analyses were performed with commercial statistical software^3^. Statistical significance was set at *p* < 0.05.

## 3 Results

### 3.1 Demographics

A total of 577 participants completed the survey, 10 of which indicated they were previously a credentialed VTS but currently were not. When these 10 participants were asked why they were no longer credentialed (allowing for more than one answer), the top reasons were, “Didn't meet recertification requirements (*n* = 4),” “Retired (*n* = 3),” and “Didn't have an incentive to recertify/was not using it (*n* = 2).”

Participants were asked to indicate their specialty academy (*n* = 577), with the largest number being members of the AVECCTN (233 [40.4%]), followed by the Academy of Veterinary Technicians in Anesthesia and Analgesia (137 [23.7%]) and the Academy of Internal Medicine Veterinary Technicians (88 [15.3%]) (Table 1). The majority of participants reported practicing in the United States (440/521 [84.5%]), followed by Canada (43/521 [8.3%]) and Australia (18/521 [3.5%]). When asked to describe the structure of their practice's ownership, the largest number (228/516 [44.2%]) reported working in a corporate setting or a privately owned practice (128/516 [24.8%]). Participants were predominantly White (455/520 [87.5%]), non‐Hispanic (436/518 [84.2%]), and women (456/520, 87.7%), aged between 30 and 49 years (Table 2). The largest number of participants reported working in the field for 16–20 years (148/577 [25.6%]) or 21–30 years (177/577 [30.7%]). When asked how long they had been a credentialed VTS, participants most commonly responded 2–5 years (168/577 [29.1%]) or 6–10 years (163/577 [28.2%]). The majority of participants reported not having a veterinary medicine or technology‐related master's degree (525/577 [91.0%]) (Table 2).

**TABLE 1:** Specialty academies reported by veterinary technician specialists (*n* = 577).

Academy of Veterinary Emergency and Critical Care Technicians and Nurses	233 (40%)
Academy of Veterinary Technicians in Anesthesia and Analgesia	137 (24%)
Academy of Internal Medicine Veterinary Technicians	88 (15%)
Academy of Veterinary Technicians in Clinical Practice	51 (9%)
Academy of Veterinary Dental Technicians	37 (6%)
Academy of Laboratory Animal Veterinary Technicians and Nurses	28 (5%)
Academy of Veterinary Surgical Technicians	26 (5%)
Academy of Veterinary Nutrition Technicians	24 (4%)
Academy of Veterinary Ophthalmic Technicians	22 (4%)
Academy of Veterinary Behavior Technicians	21 (4%)
Academy of Dermatology Veterinary Technicians	18 (3%)
Academy of Veterinary Clinical Pathology Technicians	16 (3%)
Academy of Veterinary Technicians in Diagnostic Imaging	14 (2%)
Academy of Veterinary Zoological Medicine Technicians	13 (2%)
Academy of Equine Veterinary Nursing Technicians	9 (2%)
Academy of Physical Rehabilitation Veterinary Technicians	5 (1%)

*Note*: Participants could select more than one academy.

**TABLE 2:** Demographics of 577 veterinary technician specialists (VTS).

Age (years) (*n* = 519)		Sex/gender identity (*n* = 520)		Race (*n* = 520)		Ethnicity (*n* = 518)		Years in the veterinary field (years) (*n* = 577)		Years credentialed VTS (years) (*n* = 577)	
18–29	21 (4%)	Male	47 (9%)	African American/Black	4 (1%)	Hispanic/Latinx	47 (9%)	≤5	15 (3%)	≤2	71 (12%)
30–39	191 (37%)	Female	456 (88%)	Asian	7 (1%)	Not Hispanic/Latinx	436 (84%)	6–10	65 (11%)	2–5	168 (29%)
40–49	207 (40%)	Nonbinary, nonconforming	7 (1%)	Biracial/Multiracial	9 (2%)	No response	35 (7%)	11–15	108 (18%)	6–10	163 (28%)
50–59	59 (11%)	No response	9 (2%)	Middle Eastern	2 (>1%)			16–20	148 (26%)	11–15	98 (17%)
60–69	32 (6%)	Other	1 (<1%)	Native American/Indigenous	13 (3%)			21–30	177 (31%)	16–20	48 (8%)
>69	4 (1%)			Native Hawaiian/Pacific Islander	3 (1%)			≥30	64 (11%)	≥20	29 (5%)
No response	5 (1%)			White/Caucasian	455 (88%)						
				No response	18 (4%)						
				Prefer to self‐describe	9 (2%)						

### 3.2 Reasons to Obtain VTS Credentials

**FIGURE 1:**
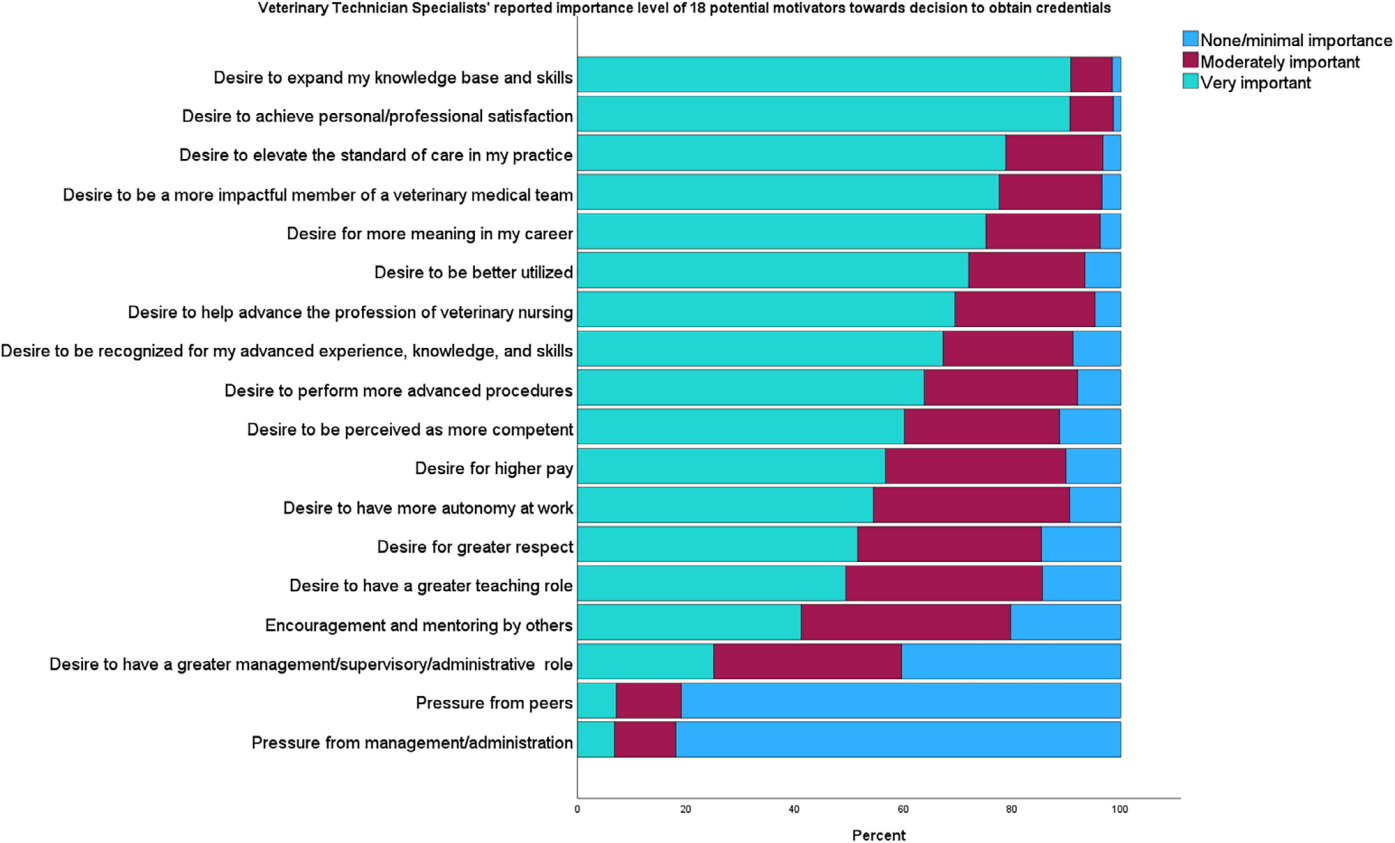
Bar graph showing the relative importance of 18 potential motivators for obtaining specialty credentials reported by 577 veterinary technician specialists.

Participants were asked to indicate the importance of 18 potential motivators toward their decision to obtain VTS credentials (Figure 1). The factors most often rated as “very important” included “Desire to expand knowledge base and skills” (90.8%), “Achieve personal/professional satisfaction” (90.6%), “Elevate the standard of care within the practice” (78.8%), and “Be a more influential member of the veterinary team” (77.6%).

Factor analysis identified four items that explained 58.4% of the variance in participant responses associated with reasons to become VTS credentialed, including expanded responsibilities (*α* = 0.77, *Ω* = 0.77), recognition/respect (*α* = 0.81, *Ω* = 0.81), personal growth (*α* = 0.75, *Ω* = 0.76), and external influences (*α* = 0.69, *Ω* = 0.72), corresponding to 30.68%, 13.64%, 8.63%, and 5.40% of the variance, respectively (Table 3). The mean value of each factor was calculated, resulting in the following order of importance: personal growth (*x* = 2.78), recognition/respect (*x* = 2.48), expanded responsibilities (*x* = 2.43), and external influences (*x* = 1.57). When assessing differences of mean scores for the four factors based on age (≤39, 40–49, and ≥50 years), there were significant differences for recognition/respect (*p* = 0.015) and external influences (*p* < 0.001) but not personal growth (*p* = 0.05) or expanded responsibilities (*p* = 0.21). Participants ≥50 years were less likely to report that recognition/respect or external influences were important factors in their decision to become credentialed as a VTS.

**TABLE 3:** Veterinary technician specialists’ reasons for pursuing advanced credentials: Rotated component matrix of four factors (expanded responsibilities, recognition/respect, personal growth, external influences).

	Factors			
	Expanded responsibilities	Recognition/Respect	Personal growth	External influences
Desire to have more autonomy at work	0.717			
Desire to perform more advanced procedures	0.709			
Desire to be better utilized	0.690			
Desire for more meaning in one's career	0.616			
Desire to have a greater teaching role	0.521			
Desire to have a greater management/supervisory/administrative role	0.423			
Desire to be recognized for one's advanced experience, knowledge, and skills		0.830		
Desire to be perceived as more competent		0.809		
Desire for greater respect		0.744		
Desire for higher pay		0.585		
Desire to expand one's knowledge base and skills			0.772	
Desire to elevate the standard of care in one's practice			0.690	
Desire to be a more influential member of a veterinary medical team			0.671	
Desire to achieve personal/professional satisfaction			0.623	
Desire to help advance the profession of veterinary nursing			0.535	
Pressure from management/administration				0.910
Pressure from peers				0.895
Encouragement and mentoring by others				0.466

*Note*: Numbers reflect the “factor loadings” for each variable on each extracted factor after the factor rotation.

### 3.3 Impact of Obtaining VTS Credentials

**FIGURE 2:**
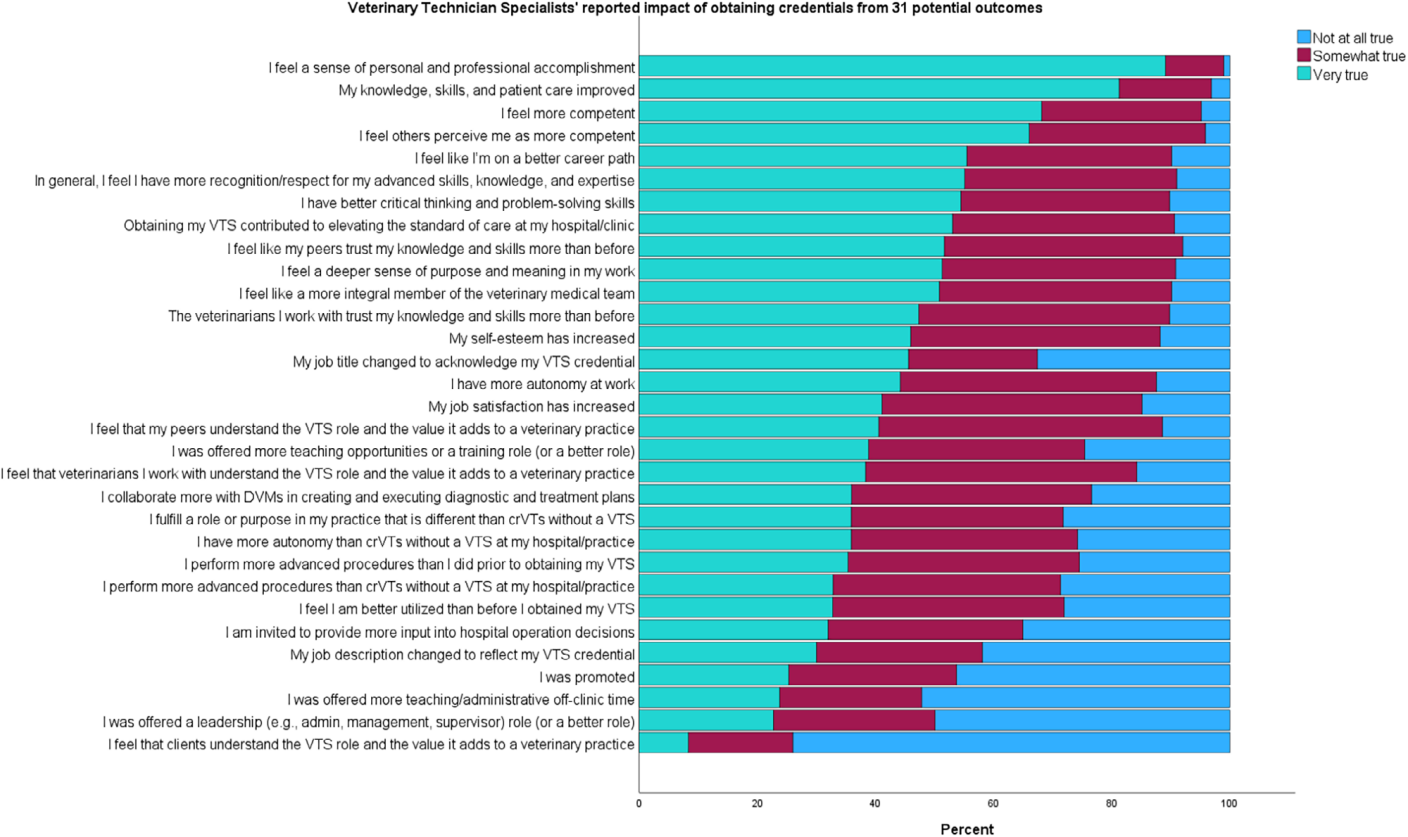
Bar graph displaying the professional impact of 31 potential outcomes from obtaining specialty credentials reported by 577 veterinary technician specialists. crVTs, credentialed/registered veterinary technicians; DVM, veterinarian; VTS, veterinary technician specialist.

Participants were asked to indicate the effects of obtaining VTS credentials by assessing how true 31 potential outcomes were for them (not true at all, somewhat true, very true). The changes endorsed as “very true” most frequently included, “Feeling a sense of personal and professional accomplishment” (89.1%), “Knowledge, skills, and patient care improved” (81.3%), and “Feeling more confident” (68.1%) (Figure 2).

Factor analysis of the 31 potential results of VTS credentialing resulted in five items that explained 56.68% of the variance, including expanded responsibilities (*α* = 0.886, *Ω* = 0.887), professional recognition/respect (*α* = 0.863, *Ω* = 0.862), career growth (*α* = 0.850, *Ω* = 0.849), elevated role (*α* = 0.652, *Ω* = 0.658), and personal growth (*α* = 0.759, *Ω* = 0.768) (Table 4). These factors corresponded to 36.10%, 7.88%, 4.62%, 4.23%, and 3.85% of the variance, respectively. The mean value of each factor was calculated, indicating the most commonly endorsed changes were personal growth (*X* = 2.58) and professional recognition/respect (*X* = 2.43), followed by expanded responsibilities (*X* = 2.16), elevated role (*X* = 2.00), and career growth (*X* = 1.87). There were no differences for any of these factors based on whether someone worked in a corporate or a private practice (personal growth: *p* = 0.566; professional recognition/respect: *p* = 0.226; expanded responsibilities: *p* = 0.344; elevated role: *p* = 0.648; or career growth: *p* = 0.354).

**TABLE 4:** Professional impact of obtaining advanced credentials reported by 577 veterinary technician specialists: Rotated component matrix of five factors (expanded responsibilities, recognition/respect, career growth, personal growth, elevated role).

	Factors				
	Expanded responsibilities	Recognition/respect	Career growth	Personal growth	Elevated role
Performs more advanced procedures at one's hospital/practice than CrVTs without a VTS credential	0.796				
More autonomy at one's hospital/practice than CrVTs without a VTS credential	0.749				
Performs more advanced procedures than before obtaining one's VTS credential	0.694				
Feels better utilized than before obtaining one's VTS credential	0.670				
More autonomy at work	0.538				
Fulfills a role or purpose in one's practice that is different from CrVTs without a VTS credential	0.505				
Collaborates more with DVMs in creating and executing diagnostic and treatment plans	0.491				
Obtaining VTS credentials contributed to an elevated standard of care at one's hospital/clinic	0.453				
Feels more competent as perceived by others		0.687			
In general, feels like one has more recognition/respect for advanced skills, knowledge, and expertise		0.630			
Feels like one is on a better career path		0.584			
Feels like a more integral member of the veterinary medical team		0.579			
Knowledge and skills more trusted by peers		0.559			
Knowledge and skills more trusted by the veterinarians with which one works		0.520			
Increased job satisfaction		0.515			
A leadership (e.g., administrative, management, supervisor) role (or a better role) was offered			0.805		
More teaching/administrative off‐clinic time was offered			0.743		
More teaching opportunities or a training role (or a better role) were offered			0.722		
A job promotion was received			0.655		
More input into hospital operation decisions is requested			0.590		
Job description reflects VTS credentialing			0.553		
Improved critical thinking and problem‐solving skills				0.740	
Improved knowledge, skills, and patient care				0.708	
Feels more competent				0.657	
Improved self‐esteem				0.497	
Feels a deeper sense of purpose and meaning in one's work				0.309	
Feels a sense of personal and professional accomplishment				0.332	
Feels that peers understand the VTS role and the value it adds to a veterinary practice					0.740
Feels that veterinarians with which one works understand the VTS role and the value it adds to a veterinary practice					0.615
Job title changed to acknowledge one's VTS credential					0.599
Feels that clients understand the VTS role and the value it adds to a veterinary practice					0.398

*Note*: Numbers reflect the “factor loadings” for each variable on each extracted factor after the factor rotation.

Abbreviations: CrVTs, credentialed veterinary technicians; DVM, veterinarian; VTS, veterinary technician specialist.

To gather more information about specific changes in expanded responsibilities and autonomy, participants were given a series of 14 tasks and asked to indicate any changes surrounding if, and how, they performed them before and after VTS credentialing. For 13 of these tasks, the most common response was that the task was not performed before or after VTS certification, including pericardiocentesis (420/524 [80.2%]), management of dialysis patients (394/524 [75.2%]), unblocking cats (313/525 [59.6%]), performing thoracocentesis (308/524 [58.8%]), and performing point‐of‐care ultrasound (308/526 [58.6%]) (Table 5).

**TABLE 5:** Changes in responsibilities and autonomy after obtaining advanced credentials reported by 577 veterinary technician specialists.

	Did not perform this task before VTS credentialing and still does not perform this task	Before VTS credentialing, performed this task with direct supervision	Before VTS credentialing, performed this task with no/minimal supervision	Did not perform this task before VTS credentialing but now performs this task with direct supervision	Did not perform this task before VTS credentialing but now performs this task with no/minimal supervision
Perform pericardiocentesis (*n* = 524)	420 (80%)	38 (7%)	13 (3%)	40 (8%)	13 (3%)
Manage dialysis patients (*n* = 524)	394 (75%)	49 (9%)	22 (4%)	35 (7%)	24 (5%)
Unblock cats (*n* = 525)	313 (60%)	79 (15%)	64 (12%)	42 (8%)	27 (5%)
Perform thoracocentesis (*n* = 524)	308 (59%)	74 (14%)	36 (7%)	71 (14%)	35 (7%)
Perform point‐of‐care ultrasound (*n* = 526)	308 (59%)	70 (13%)	60 (11%)	44 (8%)	44 (8%)
Conduct routine recheck and follow‐up appointments with report to DVM (*n* = 524)	289 (55%)	65 (12%)	110 (21%)	31 (6%)	29 (6%)
Perform euthanasia (*n* = 526)	285 (54%)	111 (21%)	95 (18%)	15 (3%)	20 (4%)
Recommend an initial diagnostic plan to the DVM (*n* = 527)	233 (44%)	92 (18%)	103 (20%)	63 (12%)	36 (7%)
Generate an initial patient problem list (*n* = 527)	227 (43%)	76 (14%)	163 (31%)	32 (6%)	29 (6%)
Manage ventilator patients (*n* = 527)	178 (34%)	121 (23%)	146 (28%)	43 (8%)	39 (7%)
Interpret/screen results of diagnostic blood work (*n* = 528)	140 (27%)	134 (25%)	135 (26%)	80 (15%)	39 (7%)
Design anesthetic plans (*n* = 527)	132 (25%)	127 (24%)	181 (34%)	50 (10%)	37 (7%)
Initiate and coordinate life‐saving treatments in an emergency according to standard protocol (*n* = 527)	108 (21%)	151 (29%)	154 (29%)	53 (10%)	61 (12%)
Induce, maintain, and monitor anesthesia (*n* = 527)	52 (10%)	94 (18%)	325 (62%)	21 (4%)	35 (7%)

Abbreviations: DVM, veterinarian; VTS, veterinary technician specialist.

Participants were asked about additional changes and benefits associated with obtaining VTS credentials. A total of 441 of 577 (76.4%) participants responded that they had received a pay increase, with the majority (77.4%) reporting an increase of ≤10% (≤2%: 77/439 [17.5%]; 3%–5%: 140/439 [31.9%]; 6%–10%: 123/439 [28.0%]). Most participants did not receive a monetary bonus (452/576 [78.5%]) but reported that their employer paid their annual VTS academy dues (348/576 [60.4%]). Participants who reported not having a production‐based income or a portion of their wage as production before earning VTS credentials (532/576 [92.4%]) were asked if they were offered a production‐based income (or a portion of their wage as production) after completing the VTS; 511 of 532 (96.1%) participants responded “no.” Likewise, those participants who reported not having profit sharing or stock/equity options before VTS credentialing (503/575 [87.5%]) were asked if they were offered profit sharing or stock/equity options after credentialing; 478 of 501 (95.4%) responded “no.” When asked if there is a position or role at their hospital that requires VTS credentials, 457 of 574 (79.6%) participants responded “no.”

Most participants reported that their hospital acknowledged the accomplishment of obtaining their VTS credential (410/569 [72.1%]), most commonly in the form of emails, newsletter announcements, celebration/party (e.g., cake, balloons, flowers), social media recognition, and monetary compensation (e.g., pay raise, bonus, increased continuing education allowance).

When participants were asked how their VTS credential(s) are acknowledged on a daily basis in the workplace, the most common responses included the use of name badges (313/577 [54.2%]), uniform marking (191/577 [33.1%]), website or social media (180/577 [31.2%]), and during staff orientation (117/577 [20.3%]). Sixty‐eight of 577 (11.8%) participants reported that their credentials were not acknowledged. A total of 78 of 502 (15.5%) indicated their hospital educates clients about the roles and responsibilities of VTSs, most commonly through websites or other social media posts (48/78 [61.5%]), hospital TV/videos (38/78 [48.7%]), and hospital posters and other signs (36/78 [46.2%]).

### 3.4 Barriers to Optimal Utilization

When participants were asked to indicate their agreement with the statement that “CrVT/Ns are underutilized,” the majority either somewhat agreed (189/525 [36.0%]) or strongly agreed (239/525 [45.5%]). Similarly, when asked if they agree or disagree that “VTSs are underutilized,” 139 of 525 (26.5%) somewhat agreed, and 303 (57.7%) strongly agreed.

**FIGURE 3:**
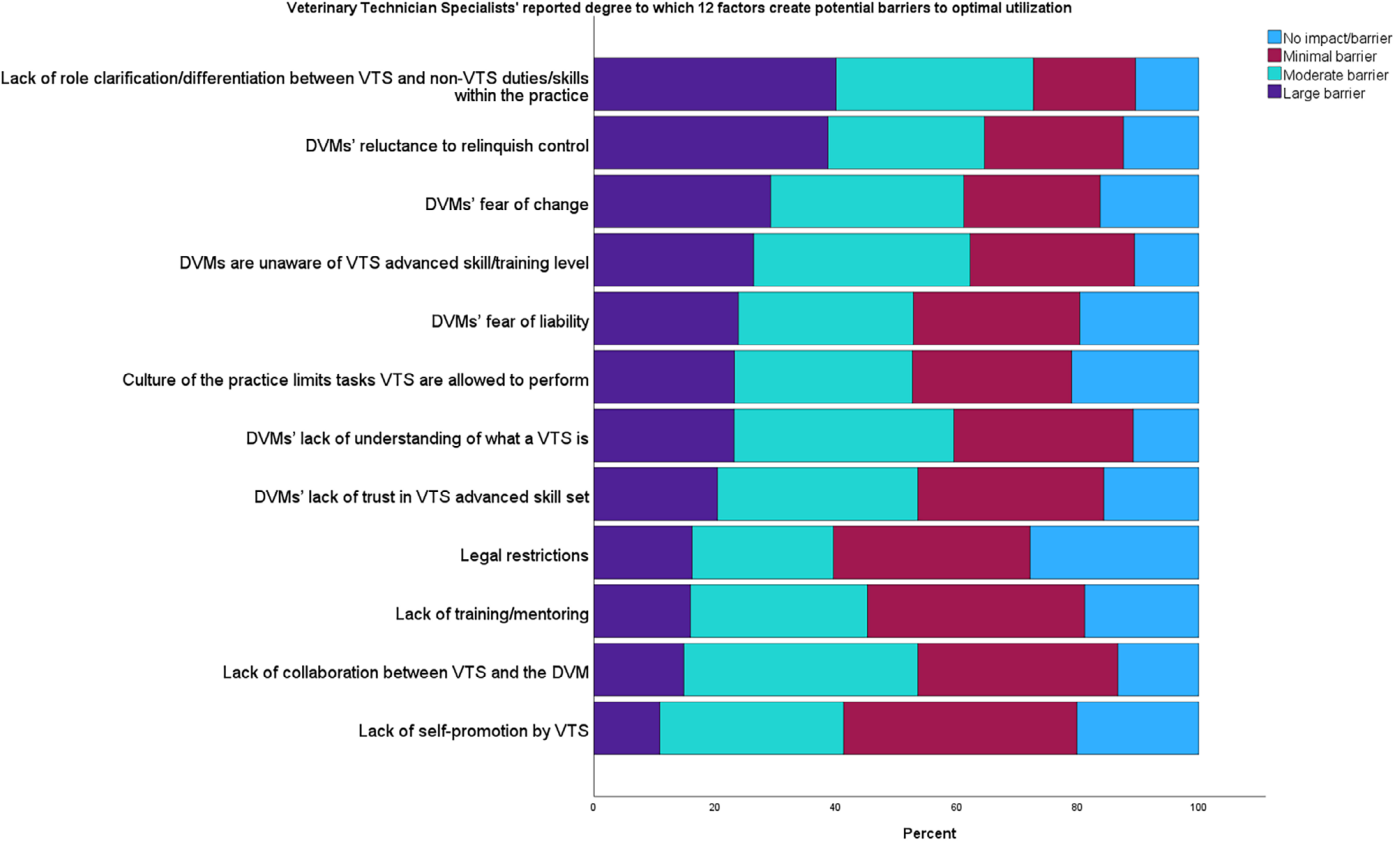
Bar graph showing the degree to which 12 factors create potential barriers to optimal utilization of veterinary technician specialists (VTS) reported by 577 VTS. DVM, veterinarian.

Participants were asked to indicate the degree to which several factors created potential barriers to optimal utilization. According to respondents, the most prominent barriers included “Lack of role clarification/differentiation between VTS and non‐VTS duties/skills within the practice” (large barrier: 221/553 [40.0%]), “Perceived veterinarians’ reluctance to relinquish control” (large barrier: 214/553 [38.7%]), and “Perceived veterinarians’ fear of change” (large barrier: 161/553 [29.1%])(Figure 3).

### 3.5 ALVP

When asked if they feel that the VTS credential could be viewed as a potential natural bridge between CrVT/Ns and an ALVP, 148 of 525 (28.2%) participants somewhat agreed, and 292 (55.6%) strongly agreed. When asked to indicate their agreement with the statement, “As a VTS, I currently function like an ALVP, with the exception of performing functions that are limited to veterinarians by state practice acts (diagnosis, prognosis, prescription, or performing surgery),” 156 of 525 (29.7%) participants somewhat agreed, and 125 (23.8%) strongly agreed. When asked whether, “As a VTS, I currently function like an ALVP and frequently collaborate with veterinarians to provide input on diagnosing, prognosing, prescribing, or performing surgery, with the veterinarian having the final sign‐off,” 129 of 525 (24.6%) somewhat agreed, and 119 (22.7%) strongly agreed.

Participants were asked if they were interested in becoming an ALVP, and 360 of 524 (68.7%) reported “yes,” while 164 (31.3%) responded “no.” There was a difference based on age (*p* < 0.001) whereby participants ≤39 years were more likely to report being interested in becoming an ALVP (166/212 [78.3%]) compared with those 40–49 years (138/207 [66.7%]) or those ≥50 years (47/94 [50%]).

Participants who reported that they were interested in becoming an ALVP were asked if they would still be interested if it required obtaining a master's degree; 183 of 357 (51.3%) responded “yes,” 58 (16.2%) said “no,” and 116 (32.5%) said they were “unsure.” When asked if a master's degree were required to become an ALVP, whether VTS credentialing should count toward the requirements of such a degree, the majority (408/519 [78.6%]) agreed, 26 of 519 (5.0%) disagreed, and 85 (16.4%) were neutral or had no opinion. There was no difference based on age for reported interests in becoming an ALVP if it required a master's degree (*p* = 0.119) or if VTS credentials should count toward the requirements of a master's degree (*p* = 0.939).

Participants were asked to choose from a list of six types of working relationship with a veterinarian they envision as most appropriate for an ALVP. The most common response was, “Indirect supervision (veterinarian available by phone/telehealth), report abnormal findings/plan to veterinarian, collaborative sign‐off on complicated cases, veterinarian check only when ALVP/veterinarian not comfortable; otherwise veterinarian reviews” (169/514 [32.9%]), followed by, “Direct supervision (veterinarian readily available on premises), report findings/plan to veterinarian, collaborate for case sign‐off with brief veterinarian check of patient/client” (158/514 [30.7%]). The least common responses were, “Independent care provider, ALVP/veterinarian have level of trust/standing orders such that only the most complicated of cases are reviewed by veterinarian” (34/514 [6.6%]) and “Immediate, direct supervision (veterinarian in visual and audible proximity), hands‐on collaboration on every case” (22/514 [4.3%]).

Participants were given an opportunity to provide comments about ALVPs and in general were more supportive of than opposed to the concept; however, many included notable caveats and concerns. Standardized credentialing and improved skill and knowledge utilization of existing CrVT/Ns and VTSs were stressed by many participants as a priority before pursuing further development of an ALVP role. Those who opposed the ALVP concept expressed skepticism that veterinarians would fully utilize ALVPs due to a perception of historic underutilization of professionals’ skills and knowledge in current roles (CrVT/Ns and VTSs), as well as the belief that elevating the roles of CrVT/Ns and VTSs is currently more important.

### 3.6 Future Plans

When participants were asked if they picture themselves as a VTS 5 years in the future, the majority (467/527 [88.6%]) reported “yes.” When those who said “no” were asked to clarify their replies, imminent retirement was the most common reason. Other common reasons included lack of recognition, not feeling valued, challenges with management or leadership, or moving into a new career role. When queried, the majority of participants reported they would recommend VTS credentialing to others (509/550 [92.5%]). When those who indicated they would not recommend VTS credentialing to others were asked to elaborate, the most common response(s) centered around the lack of changes in responsibilities, pay, and role. Personal growth, knowledge advancement, better patient care, career opportunities and potential for advancement, and advancement of the veterinary technician/nurse profession were the primary reasons why participants indicated they would recommend VTS credentialing.

## 4 Discussion

Before the current study, the motivations of a VTS and the influence of their choice to pursue the credential were only anecdotally understood. When examining the four all‐encompassing reasons why people choose to pursue VTS credentials, we found that opportunity for personal growth was the most prominent reason, specifically the desire to expand one's individual knowledge and skills and the desire to achieve personal and professional satisfaction (both rated as very important by 91% of participants). The desire to elevate their practice's standard of care (rated as very important by 79% of participants) and being a more essential member of the team (rated as very important by 78%) were also highly rated.

The next two reasons to pursue a VTS credential following the opportunity for personal growth included expanded responsibilities, such as the ability to perform more advanced procedures, enhanced clinical autonomy, elevation into a management/supervisory role, and improved utilization, and recognition/respect, comprised by the desire to be perceived as more competent and recognized for one's advanced experience and knowledge. In contrast, external factors such as pressure from peers or management appear to minimally affect the decision to pursue a VTS credential.

It is important to evaluate how professionals’ situations change after obtaining the VTS credential. When potential impacts of VTS credentialing were grouped into major categories, the largest number of respondents endorsed personal growth and recognition/respect (in alignment with motivators for pursuing the credential). The three most highly endorsed statements all related to personal growth; 89% of respondents reported it was “very true” that they feel a sense of personal and professional accomplishment associated with the VTS credential, 81% reported it was “very true” that their knowledge, skills, and patient care improved after achieving the credential, and 68% reported it was “very true” that they felt more competent after earning the VTS credential. The three next most common endorsements all related to recognition/respect and included feeling that “others perceive them as more competent” (rated as very true by 66%), feeling they are “on a better career path” (rated as very true by 56%), and that they have gained “more recognition and respect for their advanced skills, knowledge, and expertise” (rated as very true by 55%).

It would appear that the primary reasons for obtaining VTS credentials, namely personal growth and recognition/respect, are realized by many individuals. Yet, this only tells part of the story. Another key motivating factor for pursuing VTS credentials was the desire for expanded responsibilities, evident in the replies focusing on this concept. For example, 72% of participants noted the desire to be better utilized, 64% reported the desire to perform more advanced procedures, and 54% expressed the desire for more autonomy at work as “very important” motivators. Only a minority of respondents indicated that it was “very true” that they perform more advanced procedures than CrVT/Ns without a VTS credential at their hospital/practice (33%), have more autonomy than CrVT/Ns without a VTS credential at their hospital/practice (36%), perform more advanced procedures than they did before obtaining their VTS credential (35%), feel that they are better utilized than before they obtained their credential (33%), fulfill a role or purpose in their practice that is different than CrVT/Ns without a VTS credential (36%), or collaborate more with DVMs in creating and executing diagnostic and treatment plans (36%).

Although most participants (72%) reported that their hospitals acknowledged the attainment of their credential through an announcement or celebration, significant monetary rewards were less common. Social and relational recognition is likely appreciated but offers limited long‐term incentives or quality of life improvement for VTS credential holders. Although 76% of participants noted that they received a pay increase after receiving their credential, 49% of these individuals reported the raise was ≤5%, and the majority received ≤10%. In addition, most participants (78%) reported not receiving a bonus or being given the opportunity for production‐based income (92%) or profit sharing or stock/equity options (88%).

Taken together, these results suggest that for a majority of VTS holders, little substantial change in terms of expanded responsibilities, clinical autonomy, utilization, or monetary rewards currently results from VTS credentialing. A recent study found that VTSs were more likely to report ≤50% of their skills were utilized compared with CrVT/Ns [6], and only 14% reported feeling that most of their skills were utilized [6]. CrVT/Ns’ ability to positively affect medical quality and animal well‐being is an intrinsic motivator that is closely linked to the optimal skill and knowledge utilization of their education and clinical skills. However, in the current study, participants indicated that 13 of 14 advanced clinical tasks were not commonly performed either before or after earning a VTS credential.

In another recent study, the addition of VTS status did not correlate to any additional variance in job satisfaction or individual engagement [5]. The relationship between clinical qualifications and how VTSs are leveraged within the practice may help explain why one third of study participants indicated they were not interested in becoming an ALVP. Having earned VTS credentials but seeing little in the way of tangible change in clinical autonomy or task delegation, it may be difficult for VTSs to believe that an additional credential or further education would positively influence their compensation or utilization.

Optimal skill and knowledge utilization by allowing CrVT/Ns to perform at the high end of their education and credentials is not only critically important but is one of the American Veterinary Medical Association's recommendations to help address the shortage of veterinarians [8]. There are likely myriad reasons why VTS credentialing does not lead directly to expanded responsibilities and optimal utilization. When participants were asked about their perception of barriers, “Lack of role clarification/differentiation between VTS and non‐VTS duties/skills within the practice” and the perception of “veterinarians’ reluctance to relinquish control” were prominently ranked. Instead of viewing these as separate barriers, we suggest it is probable that the perception that veterinarians appear reluctant to relinquish control stems directly from confusion regarding the education, training, and credentials of various individuals in a given practice and poor differentiation between VTSs and non‐VTSs. This is further supported by the fact that only 38% of participants feel that veterinarians understand the VTS role and its value. In one recent study, only 40% of veterinary support staff reported that roles are always differentiated based on credentials within their hospital [6]. Almost 80% of study participants indicated that there are no roles in their practice that require a VTS credential, indicating that development across the industry would be beneficial to those wishing to extend their career paths after obtaining VTS credentialing.

Veterinarians may feel more comfortable delegating tasks if nondoctor labor roles are clearly delineated by name tags or uniforms. It would also be helpful to offer education to all staff members, including clinicians, about each nondoctor role and the skills and knowledge they offer. There may be value in augmenting the curricula of veterinary doctoral programs to include comprehensive education about the education, training, and clinical role of veterinary assistants, CrVT/Ns, and VTSs, in addition to advocacy and best practices for compliance with state and provincial veterinary practice acts. The current study found that only a minority of hospitals (16%) educate clients about the education, role, and training of VTSs. As with nurse practitioners in human medicine [9, 10, 11], clients may feel more comfortable working directly with a VTS after being educated about their training, skills, and knowledge and when tasks are appropriately delegated by the veterinarian.

The fact that 89% of participants reported that they see themselves as a VTS in 5 years and 93% would recommend pursuing a VTS credential to others suggests that most VTSs feel satisfied with their decision to pursue the credential even though it has not resulted in significant gains in compensation or clinical autonomy. In contrast, while veterinarians who choose to become board‐certified specialists likely share a similar intrinsic drive for personal growth and knowledge, their certification leads to the opportunity to practice advanced medicine within their specialty. Board‐certified veterinarians are widely recognized as experts in the subject matter and benefit from a variety of opportunities for professional development and advancement typically unavailable to VTSs. It is time for veterinary medicine to similarly reward VTSs, not only with intrinsic personal satisfaction but also with tangible, external rewards such as financial compensation and increased clinical autonomy. The profound financial influence that CrVT/Ns have on a practice's revenue has been well documented [12, 13]. Further research is warranted that focuses on the financial value added by VTSs.

There are many changes that can be made to ensure that passionate, committed, and ambitious veterinary technicians and nurses view VTS certification as a viable career option that leads to increased job satisfaction and lower levels of burnout. The average annual turnover rate among veterinary team members is 23% and increases each year [14], with several studies suggesting that more than 50% of veterinary technicians struggle with burnout or compassion fatigue [15, 16, 17, 18]. The recent Brakke study, for example, found that 86% of veterinary technicians reported medium or high burnout [15]. Numerous studies exploring burnout among both veterinarians and CrVT/Ns have identified contributing factors that include excessive workload, on‐call duties, limited resources, workplace conflicts, euthanasia, unrealistic expectations from pet owners, moral injury, and high debt load [17, 19, 20, 21, 22, 23, 24, 25]. Work factors that appear to mitigate burnout include a positive workplace culture, autonomy and control, being a part of a team, respect, and opportunities to develop and use one's professional skills [20, 22, 26, 27, 28, 29, 30, 31]. Factors cited as protective in these studies were also cited by VTSs in the current study, indicating that a focus on healthy and positive practice culture might be effective in preventing attrition and enhancing retention across nondoctor labor roles.

A recent study by the American Animal Hospital Association found that people's decisions to remain in their position included fair compensation, doing meaningful work, flexibility in scheduling or duties, and feeling like one's work is appreciated [32]. The focus on compensation was echoed by the recent Brakke study [15], in which compensation was found to be the most significant challenge and reported as a critical issue by 68% of participants, with 59% of participants feeling little or no satisfaction with their financial situation. As a likely consequence, 25% of CrVT/Ns report working a second job outside of their veterinary practice [15], a situation that potentiates burnout. The fact that 76% of participants in the current study indicated they received a pay increase upon VTS credentialing but that half of those participants reported a raise of 5% or less clearly signifies an area of needed change. Adequate, fair compensation is needed to attract and retain talented VTSs.

There are several limitations to the current study that should be noted, including those inherent in online surveys. Our sample consisted of only a percentage of VTSs (approximately 38%), of which a disproportionate number were VTSs in emergency and critical care medicine; therefore, caution is warranted when generalizing to other VTS academies, particularly in responses related to specific skills. Another limitation is response bias; VTSs who feel strongly about the topics in this study may have been more likely to respond. There is inherent risk in asking respondents to choose answers from and rank items within lists that have been drafted and designed by the investigators, because the choices may not accurately reflect the feelings and perceptions of the participants and may inadvertently guide them to responses that may mirror the investigators’ bias. In addition, while participants were asked if a VTS credential should count toward master's degree completion, which may be required for an ALVP role, this may have been misleading because individual institutional accreditation requirements may preclude the granting of a master's degree to individual VTSs who have not completed a precursor college degree (associate's or bachelor's).

In conclusion, the current study suggests that most VTSs are happy with their choice to pursue advanced training. One of the primary motivating factors, an intrinsic desire for personal growth, is achieved by most VTSs, but unfortunately financial rewards and increased responsibility resulted less often. Considering the expanded knowledge and skill of the VTS certificate holders, increased pay and added professional responsibilities may improve retention, which could help alleviate staff shortages in veterinary medicine.

## Author Contributions

**Lori R. Kogan**: conceptualization, data curation, formal analysis, methodology, project administration, writing – original draft, writing – review and editing. **Leslie Carter**: conceptualization, writing – review and editing. **Kelly Foltz**: conceptualization, writing – review and editing.

## Conflicts of Interest

The authors declare no conflicts of interest.

## Endnotes

1 Ograin V. Personal communication, 2024.

2 Qualtrics.

3 SPSS, 28.01, IBM.
